# Predicted impact of vaccination against human papillomavirus 16/18 on cancer incidence and cervical abnormalities in women aged 20–29 in the UK

**DOI:** 10.1038/sj.bjc.6605528

**Published:** 2010-01-26

**Authors:** J Cuzick, A Castañón, P Sasieni

**Affiliations:** 1 Cancer Research UK Centre for Epidemiology, Mathematics and Statistics, Wolfson Institute of Preventive Medicine, Queen Mary University of London, Charterhouse Square, London EC1M 6BQ, UK

**Keywords:** HPV vaccination, cervical screening, cervical cancer, CIN, cytology, modelling

## Abstract

**Background:**

Human papillomavirus (HPV) vaccination has been approved in more than 90 countries and is being implemented in many of these. In the UK, vaccination for girls aged 12–13 with catch-up for girls up to age 18 was introduced in 2008, using the bivalent GSK vaccine (Cervarix).

**Methods:**

We modelled the proportion of abnormal smears, cervical intraepithelial neoplasia grade 3 (CIN3) and invasive cancer, which will be prevented in women aged 20–29 in the UK as a result of HPV vaccination.

**Results:**

It will take many years for the full benefit of vaccination to be achieved. The earliest effects will be seen in women aged 20–29. With 80% coverage in women aged 12–13, we project an eventual 63% reduction in invasive cancer, a 51% reduction in CIN3 and a 27% reduction in cytological abnormalities before age 30. The full effect in this age group will not be seen until 2025, although half of the benefit will be seen by 2019 in England, where screening starts at age 25. However in Scotland and Wales, where screening starts at age 20, 50% of the benefit for CIN3 and abnormal smears (but not cancer) will be seen earlier.

**Conclusion:**

Substantial reductions in disease can be anticipated by vaccination, but most of the benefit will not be apparent for at least another decade. High vaccine coverage is the key factor for achieving these benefits.

High-risk human papillomavirus (HPV) types have been found in more than 95% of women with invasive cervical cancer (Walboomers *et al*, 1999). The greatest proportion is caused by HPV 16, found in about 55% of these cancers worldwide, but higher in the UK and Western Europe (WHO/ICO Information Centre on HPV and Cervical Cancer, 2007) followed by HPV 18 (found in about 16%) (Smith *et al*, 2007).

To date two vaccines that protect against HPVs 16 and 18 are commercially available, Gardasil (Merck, West Point, PA, USA) and Cervarix (GSK, Brentford, London, UK), for intramuscular use in three doses over 6 months; Gardasil also protects against HPVs 6 and 11, which cause genital warts. The vaccines also differ by the type of adjuvant used. Vaccination after infection appears to have no therapeutic value for either vaccine. Several randomised trials have been reported for each vaccine. The phase III trials focused on persistent infection and cervical intraepithelial neoplasia (CIN) associated with the relevant HPV types (Harper *et al*, 2004, 2006; FUTURE II Study Group, 2007; Garland *et al*, 2007; Paavonen *et al*, 2009). Additional smaller phase II trials were primarily to evaluate immune response (Mao *et al*, 2006; Villa *et al*, 2006).

Human papillomavirus vaccination, approved in more than 90 countries, is being implemented in many. In the UK, vaccination for girls aged 12–13 with catch-up for girls up to age 18 was introduced in September 2008 using Cervarix (Table 1). The programmes in the 4 nations (England, Scotland, Wales, N Ireland) are very similar, but there are slight differences in the catch up provision. As the impact of vaccination will first become apparent in younger women, we have estimated the number of abnormal smears, CIN grade 3 lesions (CIN3) and invasive cancers, which will be prevented by HPV vaccination at ages 20–29 in the UK. Because CIN3 and abnormal smears are only relevant in screened population that in England does not include ages 20–24 (but does in Scotland, Wales and Northern Ireland), we estimate these end points, both in the presence and absence of screening in this age group.

## Materials and methods

Because vaccination is only effective in uninfected women, the impact of the catch-up programme presents difficulties in modelling, due to the higher pre-exposure to HPV at the time of vaccination. We modelled the proportion of each of the three end points that will be prevented by the UK vaccination programmes at different calendar time points. This was done separately for HPV 16 and HPV 18 for each end point.

In our model, the relative protection afforded by vaccination depends on the product of four factors:

1. The proportion of the disease end point caused by HPV types 16, 18, 31, 33 and 45.
2. The relative protection (against disease caused by HPV) afforded by vaccination before infection (this is assumed to be 100% for types 16 and 18, but lower for types 31, 33 and 45 when cross-protection is included and 0 when no cross-protection is included).
3. The relative protection in older girls compared with a girl vaccinated at age 13 (Table 2). For vaccination at age 16 (for example), this is based on the proportion of disease occurring before age 30 that is caused by infection before age 16. This is assumed to be independent of the type of HPV.
4. The likelihood of being vaccinated. This is expressed relative to the coverage in girls aged 12–13 who would be offered vaccination as part of a school-based programme (Table 2).

Factors 1 and 2 are taken to be independent of age (under 30) and calendar year. Factors 3 and 4 are determined at the time of vaccination and carried forward as the woman ages. We calculate the number of events expected in single year age groups and then present the (average) rate in combined age groups (20–24, 25–29 and 20–29).

### Assumptions

The HPV vaccine programme in the UK is primarily school based. Provisional data submitted by Primary Care Trusts in England by the 30 April 2009 report that 86.2% of girls aged 12–13 have had their first HPV jab and 82.6% have received their second jab (NHS Immunisation Information, 2009). Coverage in the catch-up cohort (much of which is not in full-time education) is much lower with 52.5% of 17- to 18-year olds receiving the first dose and 40.8% receiving the second (NHS Immunisation Information, 2009). The vaccine uptake figures (which include vaccinations from September 2008 to mid-February 2009) for Scottish girls in the second, fifth and sixth year of secondary school are 92.2% for the first dose and 87.8% for the second dose (Information Services Division NHS National Services Scotland, 2009). Uptake figures for girls that are no longer at school have not been published, but they will be substantially lower. Furthermore Australia's latest coverage report (published December 2008), including data from all three vaccine doses, suggests a 70% coverage for school-based programmes across all cohorts vaccinated (school years 7–12) (Brotherton *et al*, 2008).

On the basis of these experiences, our base case assumption is that coverage will be 80% at age 12–13 and somewhat lower at older ages (Table 2), but we also present estimates based on 70 and 100% coverage at age 12–13. The relative protection afforded by 100% coverage corresponds to the protection in vaccinated women.

#### HPV exposure before vaccination

Data from the vaccine trials indicate that vaccination does not convey protection once the subject has been exposed to the virus (Schiller *et al*, 2008). However women infected by one type are still protected against the other type. Thus, for example, women infected with HPV type 18 only before vaccination will still benefit from vaccination, but the benefit will be less than in women naive to both types at vaccination. The relative protection of vaccinating a group of women some of whom have been infected compared to a group who are still naive depends not only on the proportion infected before vaccination but also the proportion who will eventually become infected in the absence of vaccination. Thus, for instance, if 50% would be infected with HPV 16 by age 30, and 10% are already infected before vaccination, then the effect of the vaccine, against HPV 16-induced disease, is only 80% of that achieved in a naive population.

Statistics from the United States estimate that approximately 13% of women aged 15 have ever had sex (Guttmacher Institute, 2006) and a survey in the UK estimated that 26% of women had had sex by age 16 (Wellings *et al*, 2001). Based on this evidence, it is very likely that most girls aged 12–13 will be HPV naive at the time of vaccination, but that the benefit of vaccination will be attenuated in the catch-up group. We anticipate 30–40% of women to be sexually active by age 17 and around 70% by age 19 (Wellings *et al*, 2001; Guttmacher Institute, 2006). Evidence suggests that women become HPV positive shortly after they become sexually active. In a cohort of HPV-negative young women in the USA, 17% were found to be HPV positive within 12 months and 55% within 3 years of entry into the study; 59% of HPV-positive women were infected with a high-risk HPV type (Moscicki *et al*, 2001). Approximately half of those testing high-risk HPV positive (for a pool of high-risk types) will have an infection with type 16 or 18 (Hibbitts *et al*, 2008; Sargent *et al*, 2008). Furthermore a recent study found that the mean time between incident HPV infection and the development of squamous intraepithelial lesions related to HPV 16 or 18 was 3.6 years (Trottier *et al*, 2009). However, the evidence suggests that cervical cancer only rarely develops within 8 years of HPV infection so that the catch-up programme in 16- to 18-year olds may have an effect on abnormal smears and CIN rates before age 30 but is likely to have only a minimal impact for cancer before this age. We assume that there will be no effect on cancer incidence within 8 years of vaccination and that the effect thereafter will depend on the age at vaccination. Assumptions made about the protection obtained from catch-up vaccination are detailed in Table 2. We assume the relative protection given to women vaccinated within the catch-up programme will be 70% or higher (Table 2). This roughly corresponds to 15% of 17- to 18-year olds already being infected with HPV 16 or 18 and 50% becoming infected by age 30 in the absence of vaccination. The results presented are robust to even substantial changes to the assumptions in Table 2. For instance we considered the relative protection at age 17–18 to be as high as 90% or as low as 50%.

We also assume that vaccine coverage in the population as a whole (sexually active men and women of any age) will be insufficient over the next 20 years to provide any material herd immunity.

#### Cross-protection

Both vaccines have reported information regarding cross-protection (Brown *et al*, 2009; Paavonen *et al*, 2009; Wheeler *et al*, 2009). Here we model the effect of cross-protection using recent results from the PATRICIA trial, as this trial used the vaccine being offered in the UK (Paavonen *et al*, 2009). Data on 6-month persistent infection by HPV type indicate that the vaccine is 75.7% (96.1% CI 60.4–85.7) effective against type 45, 78.7% (96.1% CI 70.2–85.2) effective against type 31 and 45.7% (96.1% CI 25.1–60.9) effective for type 33. The combined effect against types 31 and 45 was 78% and this is the figure we use in our models.

Although these results are encouraging, the duration of cross-protection is unknown and may be less than for the types in the vaccine. Thus we have modelled the effect of vaccination in two ways, once assuming no cross-protection, and again taking into account the available evidence on cross-protection from PATRICIA (and assuming no waning of protection by age 30).

However it is still premature to estimate the magnitude of the effect of cross-protection on cancer incidence precisely.

### Lesions attributable to HPV 16/18

#### Invasive cancer

The percentage of all cervical cancers attributable to HPV 16/18 in Europe has been estimated at 73% (Clifford *et al*, 2006). However the recent evidence suggests that the proportion of cancer attributable to HPV types 16 and 18 is higher in younger women than overall (Bruni *et al*, 2009).

To estimate the proportion of cancers in women aged 20–29 attributable to HPV 16/18, we have used data on HPV 16 prevalence in 5-year age groups from a WHO/ICO pooled international study (de Sanjosé *et al*, 2007; Bruni and Ferrer *et al*, 2009) to adjust this proportion specifically to women aged 20–29. This study found HPV 16 prevalence in cancers among the 20–29 year olds to be 59.1% (146 out of 247) compared to 50.8% (4333 out of 8530) for the study population as a whole. For the estimates in the model we have applied the odds ratio (OR) between the proportion of 20- to 29-year-old cases with HPV 16 and the proportion of cases at all other ages with HPV 16 (OR 1.40) to the 73% of all cases with 16 and 18. This yields an estimate of 79.1% for the proportion of cervical cancers in 20- to 29-year-old women attributable to HPV 16 or 18.

To reflect a degree of cross–protection, we assume the following: 100% protection against HPV 16/18 (which accounts for 73% of cancer according to Clifford *et al*, 2006), 78% protection against HPV 31 and 45 (which together account for 6.9% (Smith *et al*, 2007) of the cancer burden in Europe) and 46% protection against HPV 33 (which account for 4.4% (Smith *et al*, 2007) of the cancer burden in Europe). We can then estimate that by taking cross-protection into account 80.4% of cervical cancers can be prevented by vaccination.

Thus, whether we adjust for the greater prevalence of HPV 16 in cancers in young women or the potential additional benefit of cross-protection we arrive at very similar figures (79.1 *vs* 80.4%) of cervical cancer in women aged 20–29 being preventable by vaccination (Table 3). If we took both effects into account, a potential reduction of 84.3% is possible, but this is speculative at this stage.

#### Cervical intraepithelial neoplasia grade 3

The proportion of CIN3 attributable to HPV 16/18 in women aged 20–29 who had CIN3 was obtained from estimates observed in the FUTURE I and FUTURE II trials using Gardasil. The data suggest that approximately 63.5% of CIN3 observed in their trials was associated with HPVs 16 and 18 (Franceschi and Clifford, 2008; Table 3).

If we estimate the degree of cross-protection afforded by the Cervarix vaccine in the same way as we did for invasive cancer, we find the following: 100% protection against HPV 16/18 (which account for 57.5% of CIN3, Smith *et al*, 2007), 78% protection against HPVs 31 and 45 (which together account for 9.4% (Smith *et al*, 2007) of the CIN3 burden in Europe) and 46% protection against HPV 33 (which account for 3.96% (Smith *et al*, 2007) of the CIN3 burden in Europe). We can then estimate that by taking cross-protection into account 70.8% of CIN3 can be prevented by vaccination.

Estimates from the PATRICIA trial from HPV naive women indicate that the vaccine efficacy against CIN3 or worse lesions regardless of the HPV type is 87% (96.1% CI 54.9–97.7), however these estimates were based on only 26 women with CIN3 or worse. Estimates for CIN2 or worse are based on 143 cases and indicate a vaccine efficacy of 70.2% (96.1% CI 54.7–80.9). Another relevant clinical end point is how many women are treated for cervical disease. The PATRICIA trial observed a 68.8% (96.1% CI 50.0–81.2%) reduction in the number of cervical excision procedures (Paavonen *et al*, 2009). Taking into account both the modelled estimate (70.8%) and these various observed protections, we use 70% as the protection against CIN3 afforded by vaccination in HPV naive women (Table 3).

#### Cytological abnormalities

Results for women aged 20–29 from the ARTISTIC trial report that 28% of borderline or mild and 60% of moderate or worse cytology outcomes were attributable to HPV 16/18 (Sargent *et al*, 2008). We have computed estimates of the total proportion of abnormal smears associated with HPV 16/18 by weighting these estimates by the proportion of high-grade (20%) and low-grade (80%) abnormalities observed in women aged 20–29 in the Cervical Screening Programme Statistical Bulletin 2002–2003 (Department of Health, 2003). Thus, we conclude that 34% of cytological abnormalities (0.28 × 0.80=22.4% plus 0.60 × 0.20=12%) in women aged 20–29 can be prevented by vaccination (Table 3).

Direct observation from the PATRICIA trial yielded a more moderate 26.3% (96.1% CI 14.7–36.4) reduction in referrals to colposcopy associated with vaccination (Paavonen *et al*, 2009).

#### Rates of invasive cancer, CIN3 and cytological abnormalities

Rates of invasive cancer and of CIN3 (carcinoma *in situ*) were obtained from the 2003 Cancer Statistics Registrations Bulletin for England (Office for National Statistics, 2003). This year was chosen because its figures reflect the screening policy before the change to the age at which women were invited for screening was raised from 20 to 25. Because the impact on CIN3 and cytological abnormalities is strongly influenced by whether screening is offered to 20- to 24-year olds, we have calculated rates for CIN3 separately for woman aged 20–24 and for those aged 25–29. For invasive cancer, we assume that results would not initially change if screening were provided at ages 20–24.

Rates of CIN3 and cancer were estimated for single year of age for women aged 20–29 (we do this because the rates of cervical cancer are much lower at age 20–23 than at 24). For CIN3 we used single year of age data from South East England for 1990–2005 (Sasieni *et al*, 2009). For invasive cancer we used the single year of age distribution from the UK in 1971–1997 (Office for National Statistics, 1999).

In the absence of vaccination, the rate of invasive cancer for women aged 20–29 is taken to be 7.2 per 100 000 per year, the rate for CIN3 in women aged 20–24 is taken to be 260 per 100 000 per year and 322 per 100 000 per year in women aged 25–29 (Table 3).

Using the Cervical Screening Programme Statistical Bulletin 2002–2003 (Department of Health, 2003), we found the rates of cytological abnormalities per 100 women screened to be 15.2 in women aged 20–24 and 11.2 in women aged 25–29 (Table 3). No cases of CIN3 or cytological abnormalities will be detected in women aged 20–24 in England since screening does not take place in this age band. Thus, we report separately what might happen in a population screened from age 20 and in a population screened from age 25 only. For the latter we assume that starting screening at age 25 will have only a small effect on the end points detected at age 25–29 compared to starting screening at age 20. This is certainly an oversimplification, because one would expect some carryover of end points that would have been detected before age 25 if screening had taken place. This is likely to lead to increased disease detection at the first screen past age 25, but there are no reliable data from which to make an adjustment. Further, the relative effect of vaccination is not affected by what happens to the underlying rate of disease detected.

## Results

**Figure 1:**
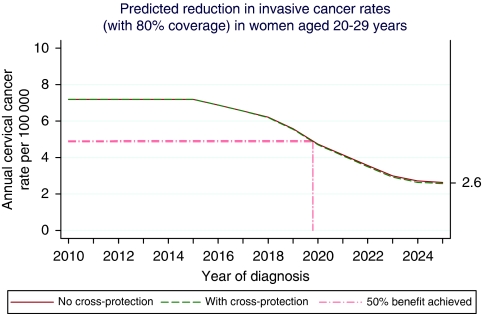
Predicted reduction in invasive cancer rates (with 80% coverage) in women aged 20–29 years.

For invasive cancer in women aged 20–29, we project a 63% reduction in rates by 2025 with 80% vaccine coverage and no cross-protection, based on an estimated 79% reduction in vaccinated women. If only 70% coverage is achieved, the reduction will be more moderate (55%). The reduction in rates taking into account cross-protection is only slightly higher. These rates are plotted against calendar year in Figure 1 and suggest that half of the total benefit on cancer rates achievable by vaccination for this age group could be achieved by the end of 2019.

**Figure 2:**
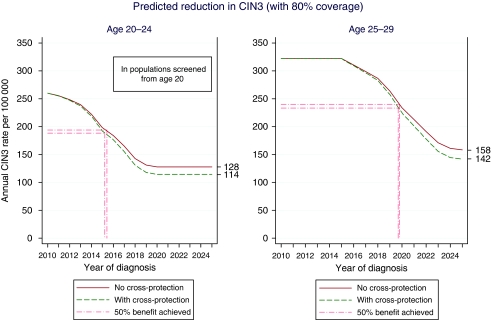
Predicted reduction in CIN3 (with 80% coverage).

We project that the CIN3 rates can be reduced by 51% with 80% coverage and no cross-protection, and by 56% taking into account the cross-protection observed in the PATRICIA trial. The reduction in rates of CIN3 due to vaccination is plotted against calendar year in Figure 2, being much more rapid at ages 20–24 than at 25–29. Assuming women aged 20–24 are screened, about half of the total benefit in this age group will be achieved by 2015, while for ages 25–29 this will occur around late 2019/early 2020.

**Figure 3:**
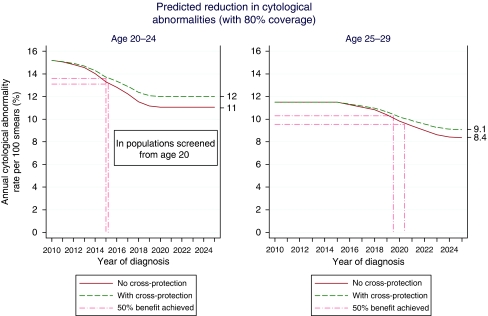
Predicted reduction in cytological abnormalities (with 80% coverage).

Without cross-protection, and assuming screening begins at age 25, we project that cytological abnormalities can be reduced by 27% with 80% coverage. If screening were to begin at age 20, we estimate that 34% of these abnormalities would be prevented. However, using more direct estimates from the PATRICIA trial, with 80% coverage a more moderate 21% reduction is projected. Reductions of cytological abnormalities as a function of calendar time are plotted in Figure 3. Thus, vaccination will reduce the rates of cytological abnormalities in women under the age of 30 in the UK, with about half of the total benefit achieved by 2015 at ages 20–24 and by late 2019/early 2020 at ages 25–29.

## Discussion

It is clear that vaccination will eventually have a substantial impact on the rates of invasive cervical cancer and its precursors. However the relative impact on abnormal cytology is much less than that observed for cervical cancer and CIN3, due to the larger impact of other HPV types on this end point. The first effects will be seen in younger women, but even then it will take 10 or more years to be appreciable, especially if screening starts at age 25. In England we will not observe the projected benefit of vaccination on precursor lesions in women aged 20–24, as the screening programme only invites women over age 25. In fact (regardless of vaccination) we are likely to observe an increase in the number of abnormal smears and CIN3 detected during the first round of screening (at age 25) compared to historical data, due to prevalent lesions that might have been detected and removed if screening started earlier. However the effect should disappear by the second round of screening.

One of the main uncertainties in modelling the impact of vaccination is coverage. We have assumed coverage of 80% as this seems likely for a school-based programme, but estimates based on 70% coverage are also given. We have assumed that vaccination offers lifetime protection, but the current analysis only requires protection for the next 18 years (i.e. up to 2025). Data on the duration of protection offered by HPV vaccines are only available up to 7.3 years after vaccination (De Carvalh and Roteli-Martin, 2009). These suggest no loss of protection, although the extent of protection remains unknown for longer periods of time and for cross-protection from HPV types not included in the vaccine.

Also, it is not clear how much efficacy will be lost if only two doses of the vaccine are received and also how many women will fall into this category. Preliminary data on antibody titres suggest two doses may be as effective as three (Dobson *et al*, 2009). However, from experience with similar vaccines, most experts believe that a single dose is unlikely to be effective; therefore women who only received one dose are probably best grouped with unvaccinated women.

Our primary model has assumed no cross-protection, but we have also evaluated a model reflecting findings on cross-protection in the PATRICIA trial. Both models take into account that previous infection with one HPV type does not weaken the protection afforded by the vaccine to other types. We used CIN3 as one of our main end points. Another relevant clinical end point would be the reduction in women treated for cervical disease, as provided for in the PATRICIA trial. However the KC65 returns used to produce screening statistics for colposcopy episodes provide only data on treatment at first appointment. Because we have been unable to estimate the baseline number of women who get treated annually in England by age, it was therefore impossible to make this calculation.

Another major unknown is whether vaccinated women will reduce their attendance for screening. This is an important issue as screening and vaccination have benefits of similar magnitude, so the benefit of vaccination on cervical cancer incidence could be fully negated if vaccinated women choose not to be screened. Furthermore, based on associations with deprivation, it is likely that girls who are not vaccinated will, as women, be less likely to be screened. Although it is possible that some cultural or religious subgroups whose lifestyle puts them at low risk will choose not to participate in either programme, it seems likely that overall women who miss out on both programmes will have a greater disease burden than would be predicted by assuming that non-compliance to one intervention is independent of non-compliance for the other. In general non-screened women have a much higher risk than screened women so reaching this group remains a priority. Screening by self-sampling based on an HPV test may help to minimise the disease burden in this group, but developing methods to achieve a high vaccine coverage in deprived groups is also important. These issues will also be important for evaluating the joint cost-effectiveness of screening and vaccination programmes (Goldhaber-Fiebert *et al*, 2008).

On a more positive note, it is likely that less frequent screening using HPV testing will become the norm, and the substantially greater level and duration of protection for this form of testing may reduce the disease burden further (Bulkmans *et al*, 2007; Cuzick *et al*, 2008; Dillner *et al*, 2008). In the long term the combination of HPV vaccination and screening for HPV DNA promises to make cervical cancer a very rare disease and eliminate the need for frequent screening and high rates of colposcopic referral.

## Figures and Tables

**Table 1:** Birth cohorts for English HPV vaccination schedule

**Academic year**							
**HPV vaccine given**	**School year 7**	**School year 8 Age 12–13**	**School year 9**	**School year 10**	**School year 11 Age 15–16**	**School year 12 Age 16–17**	**School year 13 Age 17–18**
2008/09		1 Sep 1995 to 31 Aug 1996					1 Sep 1990 to 31 Aug 1991
2009/10		1 Sep 1996 to 31 Aug 1997		1 Sep 1994 to 31 Aug 1995	1 Sep 1993 to 31 Aug 1994	1 Sep 1992 to 31 Aug 1993	1 Sep 1991 to 31 Aug 1992
2010/11		1 Sep 1997 to 31 Aug 1998					
2011/12		1 Sep 1998 to 31 Aug 1999					

**Table 2:** Estimated relative protection and coverage for vaccination at different ages relative to girls vaccinated at age 12–13

**Year of birth^a^**	**Age at vaccination (third dose)**	**Relative protection compared to those vaccinated aged 12–13^b^ (%)**	**Relative coverage compared to those aged 13^c^ (%)**
1996 or after	13	100	100
1995	15	97	95
1994	16	92	75
1993	17	77	50
1992	18	70	50
1991	18	70	50
1990 or before	Not vaccinated	0	0

No protection against invasive cervical cancer from catch-up within 8 years of vaccination.

a For example, a year of birth of 1994 means September 93 to August 94 and assumes that the majority would have a birthday by the third dose.

b Lower protection comes primarily from some teenagers being infected with either type 16 or 18 HPV before vaccination.

c Lower coverage (particularly for those born in 1991–1993) is due to the non-school-based vaccine programmes.

**Table 3:** Estimated number and rate of cancers, CIN3 and cytological abnormalities in women under age 30 in and per cent prevented by vaccination (with and without cross-protection)

	**Cancer^a^**	**CIN3^a^**		**Cytological abnormalities^b^**	
**Age group**	**20–29**	**20–24**	**25–29**	**20–24**	**25–29**
Population at risk	3 095 000^c^	1 546 000^c^	1 549 000^c^	353 239^d^	414 892^d^
Annual number of cases	224	4,013	4,996	53 672	47 914
Rate	7.2/10^5^	260/10^5^	322/10^5^	15.2%	11.2%
Per cent prevented in vaccinated women with no cross-protection	79.1%	63.5%		34%	
Number prevented with 80% coverage	142	2039	2538	14 599	13 033
Per cent prevented in women vaccinated with cross-protection	80.4%	70%		26.3%	
Number prevented with 80% coverage	144	2247	2798	11 164	9966

a Cancer Statistics Registrations 2003.

b Screening Programme Statistical Bulletin 2002–2003.

c Resident population.

d Number with an adequate smear test.
